# Adverse Drug Reaction Predictions Using Stacking Deep Heterogeneous Information Network Embedding Approach

**DOI:** 10.3390/molecules23123193

**Published:** 2018-12-04

**Authors:** Baofang Hu, Hong Wang, Lutong Wang, Weihua Yuan

**Affiliations:** 1 School of Information Science and Engineering, Shandong Normal University, Jinan 250014, China; hubaofang@sdwu.edu.cn (B.H.); wanglutong1002@163.com (L.W.); weihuayuan_qingdao@126.com (W.Y.); 2 School of Data and Computer Science, Shandong Women’s University, Jinan 250014, China; 3 Shandong Provincial Key Laboratory for Distributed Computer Software Novel Technology, Shandong Normal University, Jinan 250014, China

**Keywords:** adverse drug reaction prediction, heterogeneous information network embedding, stacking denoising auto-encoder, meta-path-based proximity

## Abstract

Inferring potential adverse drug reactions is an important and challenging task for the drug discovery and healthcare industry. Many previous studies in computational pharmacology have proposed utilizing multi-source drug information to predict drug side effects have and achieved initial success. However, most of the prediction methods mainly rely on direct similarities inferred from drug information and cannot fully utilize the drug information about the impact of protein–protein interactions (PPI) on potential drug targets. Moreover, most of the methods are designed for specific tasks. In this work, we propose a novel heterogeneous network embedding approach for learning drug representations called SDHINE, which integrates PPI information into drug embeddings and is generic for different adverse drug reaction (ADR) prediction tasks. To integrate heterogeneous drug information and learn drug representations, we first design different meta-path-based proximities to calculate drug similarities, especially target propagation meta-path-based proximity based on PPI network, and then construct a semi-supervised stacking deep neural network model that is jointly optimized by the defined meta-path proximities. Extensive experiments with three state-of-the-art network embedding methods on three ADR prediction tasks demonstrate the effectiveness of the SDHINE model. Furthermore, we compare the drug representations in terms of drug differentiation by mapping the representations into 2D space; the results show that the performance of our approach is superior to that of the comparison methods.

## 1. Introduction

Adverse drug reactions (ADRs) are side effects caused by the use of one or several drugs. Some ADRs may be part of the natural pharmacological action of a drug that cannot be avoided, but more often, they may be unpredictable at the development stage. ADRs have caused a global and substantial burden that accounts for considerable mortality and morbidity [1]. Before clinical application of a drug, it should go through two ADR detection stages, including preclinical in vitro safety profiling and clinical drug safety trials. However, since so many side effect types and drug combinations exist, many potential side effects cannot be detected during the early drug development stage [2].

Recently, with the development of data mining and computational prediction methods, researchers have collected extensive drug data from the literature, and reports and have utilized these data to predict unknown ADRs [3,4,5,6]. ADR predictions based on computational methods can point drug safety tests in the right direction and consequently shorten the time requirement and save financial costs during drug development. A large number of machine learning methods have been proposed to predict potential ADRs [7,8,9]. Vilar et al. [10] utilized known side effect information of drugs to construct an associated matrix of drugs and adverse effects and adopted the matrix completion method to predict unknown side effects. LaBrute et al. [11] processed multi-source drug target information to find association relationships between ADRs and drug targets. These prediction methods are based on single drug information, and these mined drug datasets usually contain much noise. For example, the SIDER dataset [5], which was extracted from the public ADR reports, may contain some fake or unconfirmed noise data. Researchers have established different drug databases that describe drug features from different aspects, including chemical, biological, phenotypic, and interaction relationships [4,5,6,12,13]. It is more logical to combine different drug information to reduce the prediction error. Integrating this useful complex drug information to obtain more accurate ADR predictions is more effective. Yamanishi et al. [7] used multi-source drug data from the SIDER, PubChem, DrugBank, and Matador databases to predict side effects. The prediction method they adopted was based on multiple kernel regression and canonical correlation analysis. Zhang et al. [14] integrated different drug information to calculate drug similarities and utilized the linear neighborhoods method to transform the similarities into the side effect space and predict side effects. These prediction methods are mainly for the side effects caused by a single drug. However, in real life, many patients, especially the elderly, are on multiple prescriptions to treat different diseases. Drug–drug interactions (DDIs) may change the effects of drugs and cause some potential ADRs. Therefore, predicting the potential side effects induced by DDIs is imperative. Segura-Bedmar et al. [15] utilized a text mining method to predict the occurrence of DDIs based on a shallow language learning model. Jin et al. [16] formulated the DDI type prediction problem as a multi-task dyadic regression problem and utilized the model to predict the side effect types induced by DDIs. Zhang et al. [17] collected a variety of drug data that might influence DDIs and adopted an ensemble learning method to predict the occurrence of DDIs. Motivated by the success of deep learning in many areas, Zitnik et al. [18] developed a new graph convolutional neural network for multi-relational link prediction in multimodal networks to predict the DDI types.

Although the above methods have achieved great success, the methods are mostly designed for specific ADR prediction tasks and lack generic abilities. With the development of the network embedding, learning combined characteristic embeddings of drugs has attracted great attention from researchers [19,20,21]. Every drug can be embedded into a low-dimensional feature vector, which integrates different drug information, including chemical, biological, phenotypic, and interaction relationships. The drug representations are more general and can be used for different ADR prediction tasks. Li et al. [22] proposed a matrix completion method to integrate multiple sources of drug data and predicted ADRs. Ma et al. [23] proposed a drug embedding method based on multi-view deep auto-encoders to predict ADRs. However, their works only considered immediately relevant information of side effects and neglected potential indirect information. There are some potential association relationships between different biological data. For example, drug targets propagate to another protein through the protein–protein interactions (PPI) network, because the biological function signal cascade propagates through different proteins via PPI [24,25]. When one drug acts on a known target protein, it may change another potential target protein through protein–protein interaction effects and consequently cause potential ADRs.

**Figure 1:**
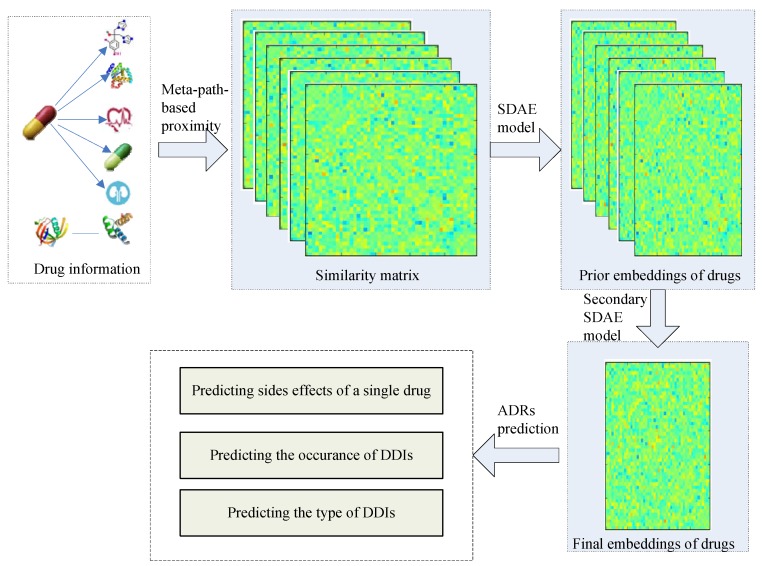
The flowchart for adverse drug reactions’ (ADRs) prediction. SDAE, stacked denoising auto-encoder; DDIs, Drug-Drug interaction.

In this work, we propose a general drug embedding method to learn the representations of drugs and predict different types of ADRs. The flowchart for ADR prediction is shown in Figure 1. We firstly modeled different drug information in a drug heterogeneous information network (drug HIN) framework and then proposed a stacking deep heterogeneous information network embedding approach based on semantic meta-paths. The generated drug embedding integrates multi-source drug information and multi-relationship side effect information to improve the ADR prediction accuracy. Especially, we utilized the target propagation strategy to recognize the potential drug targets and improve the prediction accuracy. At the target propagation stage, we need to search for the proteins that are more obviously affected by the known targets of the drug. Finding the nearest node of one node is challenging because tens of thousands of nodes exist in the PPI network. We propose using transition probability based on the random walk procedure [26,27], which is common in recommendation systems, to calculate the target propagation proximity and reconstruct the drug-target network based on the target propagation meta-path.

## 2. Datasets and Method

### 2.1. Datasets

We collected six types of drug data from seven public databases.

- Drug-Drug interaction information (DDI): Tatonetti [12] mined side effects induced by DDIs from the FDA Adverse Event Reporting System (FAERS, http://www.fda.gov/cder/aers/default.htm) and developed a database called ”TWOSIDES”. The database contains 645 drugs and ADRs caused by 63,473 combinations of different drugs.
- Protein-Protein interaction data (PPI): We downloaded the PPI network data from the Human Protein Reference Database (HPRD, http://www.hprd.org). The dataset contains 9519 proteins and 37,062 protein-protein interactions.
- Other drug information: We also obtained other drug information from four online drug information databases (DrugBank [4], the PubChem Compound database [6], the SIDER database [5], and the OFFSIDES database [12]). DrugBank is a widely-used public drug information database. From the DrugBank database, we collected drug target protein and disease treatment information. The PubChem system generates a binary substructure fingerprint for chemical structures. From the PubChem database, we searched every drug’s chemical substructure. We also extracted drug side effect information from the SIDER and OFFSIDES databases. These two databases include most associations between drugs and side effects, and we integrated the drug-side effect data obtained from the two databases.

We mapped drug ids in the TWOSIDES dataset to the other aforementioned datasets and finally constructed an integrated dataset that contained multi-source drug information, including DDIs, drug chemical substructures, drug targets, drug side effects, drug treatment, and PPI. The dataset used in this work is shown in Table 1. In this article, we did not consider the probabilities of ADR events. If a type of ADR occurred, the corresponding element in the DDI dataset or side effect dataset was labeled 1.

### 2.2. Drug HIN

**Figure 2:**
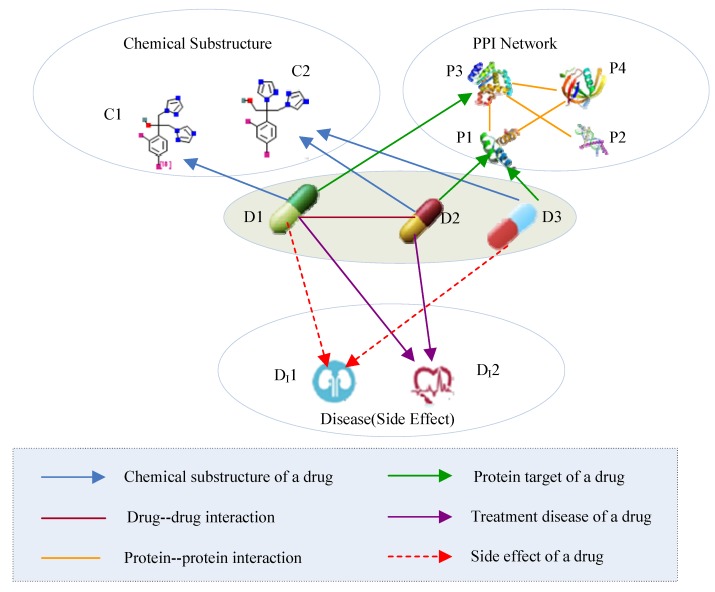
Heterogeneous drug information. PPI, protein-protein interaction.

Multi-source drug information describes different aspects of drugs and forms a typical heterogeneous information network (HIN). An HIN is a network that contains multiple types of objects or multiple types of relationships [28]. The drug HIN consists of five types of objects: drug (*D*), chemical substructure (*C*), protein (*P*), side effect (*S${}_{E}$*), and disease (*D${}_{I}$*). The five types of objects are connected through six types of links (as shown in Figure 2). A drug-drug link indicates a type of drug-drug interaction, whereas the link between a drug and its chemical substructure indicates that the drug consists of some type of chemical substructure. In Table 2, we present the semantics of the different link types in the drug HIN.

**Figure 3:**
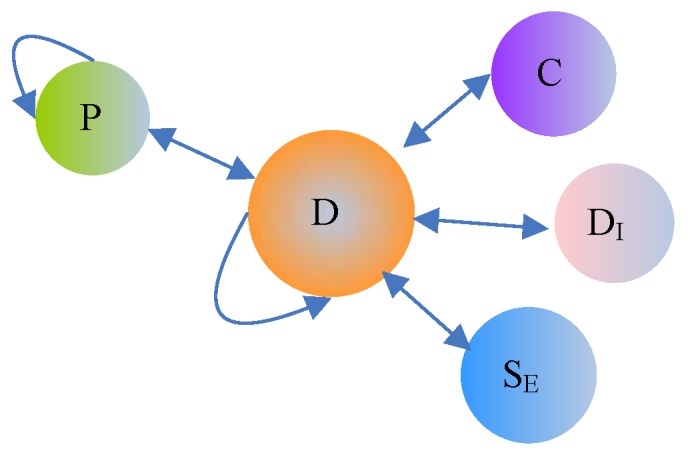
Meta-paths in drug HIN.

In HINs, two objects connect via different link types, which are called semantics meta-paths [29,30]. Given an HIN, a meta-path is a sequence of objects connected by different link types. Different types of meta-paths in the drug HIN are shown in Figure 3. Because our final goal is to learn drug representations, we only consider the meta-paths in which the starting objects are all drugs. The detailed meta-paths used in this study are summarized in Table 3.

### 2.3. Stacking Deep HIN Embedding

Our goal is to learn the low-dimensional vector representations of drugs that highly summarize the drug information and preserve the original proximity of drugs in different drug relationships, and then to predict the different types of ADRs. In this work, we proposed a semi-supervised deep model SDHINE to perform HIN embedding; the framework of the model is shown in Figure 5. In detail, first, we defined the meta-path-based proximities and constructed several homogeneous sub-networks based on the defined proximities. Then, we adopted semi-supervised stacked denoising auto-encoders (SDAE) to encode each sub-network. The supervision information is the meta-path-based proximity in every sub-network. Next, we concatenated the drug embeddings together and further learned the final drug embeddings through the secondary encoding process.

#### 2.3.1. Meta-Path-Based Proximity

We defined three types of meta-path-based proximities and constructed corresponding sub-networks based on the defined proximities.

- Constructing drug-drug interaction sub-network.

For the Drug-Drug (DD) meta-path shown in Table 3, in which the nodes are all drug nodes, we utilized the Jaccard similarity as the edge weights to construct the Drug-Drug interaction sub-network. Notably, we do not consider the interaction types at the drug embedding stage. The proximity between drug *i* and drug *j* based on meta-path DD can be calculated as shown in Equation (1).

(1) $$s\left(i,j\right)=\frac{\left|D_{i}⋂D_{j}\right|}{\left|D_{i}⋃D_{j}\right|}$$

where $D_{i}$ is a column vector with 0 and 1 elements that represent the Drug-Drug interactions between the *i*th drug and other drugs.

- Constructing sub-networks using PathSim proximity.

The meta-paths Drug-Chemical-Drug (DCD), Drug-Protein-Drug (DPD), $DD_{I}D$, and $DS_{E}D$ in drug HIN contain different types of nodes and path semantic information. For example, meta-path $D_{1}C_{1}D_{2}$ indicates that drug $D_{1}$ and drug $D_{2}$ have the same chemical substructure $C_{1}$ and there is a path from $D_{1}$ to $D_{2}$ via $C_{1}$. Therefore, we adopted PathSim [29] as the proximity measure in these meta-paths. The PathSim proximity S(i,j) is defined in Equation (2).

(2) $$s\left(i,j\right)=\frac{2\times \left|\left\{p_{i\to j}:p_{i\to j}\in P\right\}\right|}{\left|\left\{p_{i\to i}:p_{i\to i}\in P\right\}\right|+\left|\left\{p_{j\to j}:p_{j\to j}\in P\right\}\right|}$$

where $p_{i\to j}$ is a path instance between *i* and *j*, $p_{i\to i}$ is a path instance between *i* and *i*, and $p_{j\to j}$ is a path instance between *j* and *j*.

The proximities of drugs under meta-paths DCD, DPD, $DD_{I}D$, and $DS_{E}D$ (as shown in Table 3) are directly calculated using PathSim. Then, we constructed corresponding sub-networks using the proximities as the edge weights to form corresponding sub-networks.

- Reconstructing the drug-target sub-network using the target propagation method.

One innovation of our proposed approach is calculating target protein transition probabilities based on the PPI network and reconstructing the drug-target sub-network. As previously mentioned, potential association relationships exist between different biological data, especially for drug target information. When one target protein is activated by a drug, another potential protein may be activated by protein-protein interactions and consequently cause an unreported ADR. Therefore, we should reconstruct the drug-target sub-network using the target propagation strategy according to the meta-path $DP^{\left(n\right)}D\left(n\geq 2\right)$. The target propagation in the meta-path $DP^{\left(k\right)}D$ can be seen as a random walk procedure. A walker walks on the PPI network and achieves the destination protein via k−1 steps. Suppose node transition probabilities in the PPI network converge after *n* steps. The global proximity based on meta-paths $DP^{\left(n\right)}D\left(n\geq 2\right)$ is:

(3) $$\begin{array}{c} s\left(i,j\right)=\sum_{k=2}^{n}S^{\left(k\right)}\left(i,j\right)=\sum_{k=2}^{n}{\left(DP^{\left(k\right)}D\right)}_{ij} \end{array}$$

**Figure 4:**
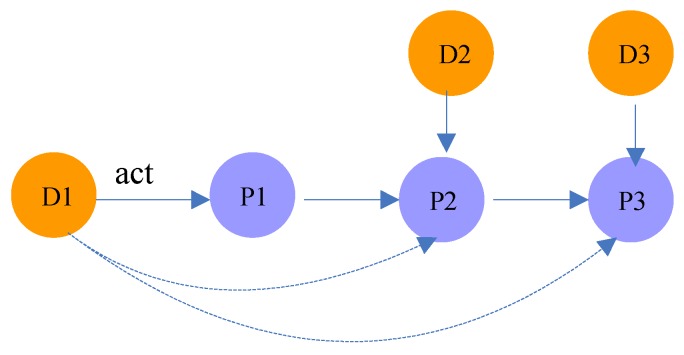
Illustration of meta-path $DP^{\left(n\right)}D$.

**Figure 5:**
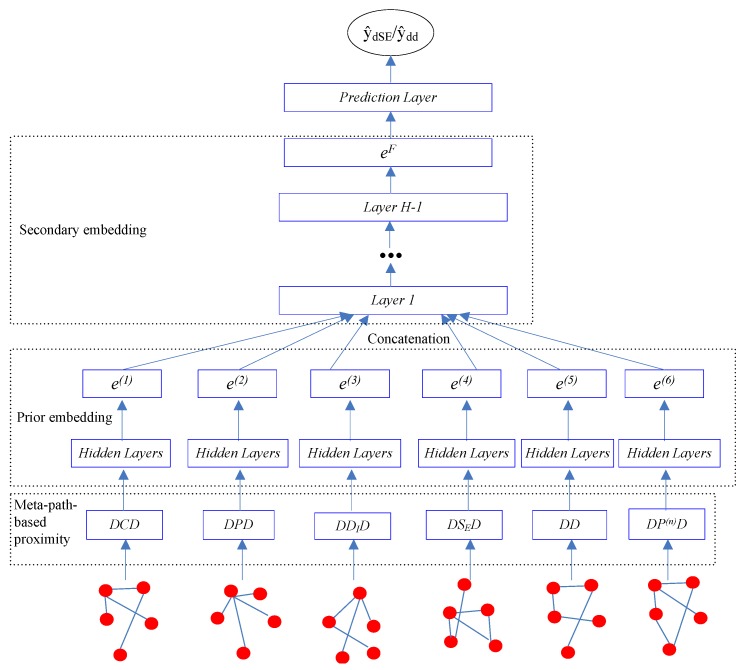
The framework of our proposed semi-supervised deep model SDHINE.

In random walk theory, the *k*-step random walk transition probability is the *k*th power of the transition probability matrix *P*. For example, as shown in Figure 4, the probability that drug $D_{1}$ acts on protein $P_{3}$ is equal to the product of the transition probability from $P_{1}$ to $P_{2}$ and the probability from $P_{2}$ to $P_{3}$.

The proximity between drug *i* and drug *j* based on meta-path $DP^{\left(k\right)}D$ can be unfolded as follows:

(4) $$\begin{array}{c} s^{\left(k\right)}\left(i,j\right)={\left(DP^{\left(k\right)}D\right)}_{ij}={\left(DP^{k}D\right)}_{ij}=D_{i}PP^{k-2}{\left(D_{j}P\right)}^{T}=D_{i}P\times \underset{k-2}{\underset{︸}{P\times P\times \cdots \times P}}\times {\left(D_{j}P\right)}^{T} \end{array}$$

where *P* is the transition probability matrix in the PPI network, *D* is the original drug-target matrix, and $D_{i}P$ represents the target row vector of the *i*th drug.

(5) $$\begin{array}{c} s\left(i,j\right)=\sum_{k=2}^{n}S^{\left(k\right)}\left(i,j\right)=\sum_{k=2}^{n}{\left(DP^{k}D\right)}_{ij}=D_{i}P\left(\sum_{k=2}^{n}P^{k-2}\right){\left(D_{j}P\right)}^{T} \end{array}$$

Given tens of thousands of nodes in the PPI network, calculating $P^{k}$ is very difficult. However, $P^{k}$ is very common in the random walk theory. During the random walk procedure, a walker starts from an initial node and moves to neighbors with probability μ and back to the initial node with probability 1−μ. Based on the Katz model [31], which is a method of computing similarities between nodes in a graph, taking into account not only the direct edges but also the indirect edges, Equation (5) can be rewritten as follows:

(6) $$\begin{array}{c} s\left(i,j\right)\approx \;D_{i}P\left[\sum_{k=2}^{n}{\left(\mu P\right)}^{k-2}\right]{\left(D_{j}P\right)}^{T}=D_{i}P\left[{\left(I-\mu P\right)}^{-1}-I\right]{\left(D_{j}P\right)}^{T} \end{array}$$

where *I* is the identity matrix and the damping factor μ usually is 0.98. The inverse of the matrix in Equation (6) can be calculated using the SVD-based matrix factorization method.

#### 2.3.2. Prior Drug Embedding

A stacking auto-encoder is a multi-layer deep neural network based on layer-wise training in which different multi-granularity data features are learned layer by layer and higher complex features are learned in higher layers. To enhance the robustness of sub-network embedding, we adopted stacked denoising auto-encoders (SDAE) in which the input neurons in every layer were randomly discarded by assigning some of the input neurons to 0 with a certain probability.

Traditional SDAE is an unsupervised model, which is composed of the encoder stage and decoder stage. At the encoder stage, the input data $x_{i}$ are mapped into representation vector space, whereas at the decoder stage, the output data ${\hat{x}}_{i}$ are the reconstructed data from $x_{i}$. The optimizer objective function of the SDAE is to minimize the reconstruction error of the output and input. The loss function is shown as follows:

(7) $$L_{1}=\sum_{i=1}^{n}{∥{\hat{x}}_{i}-x_{i}∥}_{2}^{2}$$

Here, to protect the meta-path-based proximity of every sub-network, we adopted a semi-supervised SDAE framework [32]. The different meta-path-based proximities are the supervision information that preserves the proximity of the representation of two nodes. The optimizer objective function for this goal is defined as follows:

(8) $$L_{2}=\sum_{i,j=1}^{n}{S^{\left(p\right)}}_{ij}{∥{e^{\left(p\right)}}_{i}-{e^{\left(p\right)}}_{j}∥}_{2}^{2}$$

where ${S^{\left(p\right)}}_{ij}$ is the proximity of drug *i* and drug *j* based on meta-path *p* and ${e^{\left(p\right)}}_{i}$ is the embedding of $x_{i}$ based on the corresponding meta-path.

The objective function of the semi-supervised SDAE model, which combines Equations (7) and (8), is as follows:

(9) $$L=L_{1}+\alpha L_{2}+\beta L_{reg}$$

where $L_{reg}$ (as shown in Equation (12)) is an L2-norm regularizer term to prevent overfitting and α and β are hyperparameters. $W^{\left(k\right)}$ and ${\hat{W}}^{\left(k\right)}$ are the *k*th layer weight matrices at the encoder and decoder stages, respectively.

(10) $$L_{reg}=\sum_{k=1}^{K}\left({∥W^{\left(k\right)}∥}_{F}^{2}+{∥{\hat{W}}^{\left(k\right)}∥}_{F}^{2}\right)$$

#### 2.3.3. Secondary Drug Embedding

After obtaining the sub-network embeddings, we concatenated the embeddings together and used the secondary semi-supervised stacking denoising auto-encoder to obtain the final drug embedding. Given the embedding node ${e^{\left(p\right)}}_{i}$ of drug *i* in a different meta-path *p*, we concatenated them to obtain a new representation vector $e_{i}$. Then, we utilized auto-encoder layers to learn the final embedding ${e^{F}}_{i}$ of drug *i* (as shown in Equation (11)).

(11) $$\begin{array}{c} {e^{F(1)}}_{i}=\sigma \left(W^{F(0)}e_{i}+b^{F(0)}\right)\;\\ {e^{F(2)}}_{i}=\sigma \left(W^{F(1)}{e^{F(1)}}_{i}+b^{F(1)}\right)\;\\ \ldots \;\\ {e^{F}}_{i}={e^{F(h)}}_{i}=\sigma \left(W^{F(h-1)}{e^{F(h-1)}}_{i}+b^{F(h-1)}\right) \end{array}$$

Here, we continued to adopt a semi-supervised SDAE framework to protect the original proximities of drugs in every sub-network. The supervision information in the optimizer objective function is defined as follows:

(12) $$L_{2}=\sum_{i,j=1}^{n}\frac{\sum_{p=1}^{K}\alpha^{\left(p\right)}{S^{\left(p\right)}}_{ij}}{\sum_{p=1}^{K}\alpha^{\left(p\right)}}{∥{e^{F}}_{i}-{e^{F}}_{j}∥}_{2}^{2}$$

where ${S^{\left(p\right)}}_{ij}$ is the proximity of drug *i* and drug *j* based on meta-path *p* and $\alpha^{\left(p\right)}$ is a hyperparameter, which is the weight coefficient of the meta-path *p*. At the experimental stage, the best hyperparameters $\alpha^{\left(p\right)}$ are learned using 10-fold cross-validation on 10% labeled data with a grid search over $\alpha^{\left(p\right)}\in \left\{0.1,0.2,0.3,0.4,0.5\right\}$.

The objective function of this part is shown in Equation (13).

(13) $$L_{G}=\sum_{i=1}^{n}{∥{\hat{e}}_{i}-e_{i}∥}_{2}^{2}+\alpha L_{2}+\beta L_{reg}$$

### 2.4. Prediction Formulation

For prediction tasks, learning a classifier that can be generalized to unknown ADRs is desirable. We predict the labels on training data using a fully-connected layer $y=h\left({e^{F}}_{i}\right)=\sigma \left(W^{P}{e^{F}}_{i}+b\right)$. The prediction loss is formulated by Equation (14).

(14) $$L=\sum_{y\in Y_{tarin}}\left[-y\ln y^{\prime }-\left(1-y\right)\ln\left(1-y^{\prime }\right)\right]+\lambda {∥W^{P}∥}_{F}^{2}$$

## 3. Experiment

### 3.1. Implementation and Evaluation Strategy

Proposed model: We implemented the proposed model with TensorFlow 1.2 and trained the model using the adaptive learning rate optimizer Adam [33]. All neurons were activated by the sigmoid function. We optimized the hyperparameters in the model using validation data and then fixed them for all denoising auto-encoder layers.

Baseline: In addition, we implemented the following three network embedding baselines for comparison:

- Concatenate drug features: This method is a simple original HIN embedding method [28]. The approach constructs a feature vector for each drug by concatenating the PCA representation of each correlation matrix, which represents one aspect of the drug character.
- GraphCNN [34]: GraphCNN is a recently-proposed network embedding method based on spectral convolutional operation and achieves state-of-the-art performance on important prediction problems in recommender systems. Here, first, we linearly integrated similarity matrices based on all meta-paths except the target propagation meta-path $DP^{\left(n\right)}D$ and then learned the drug embeddings using the same GraphCNN structure described in [35].
- metapath2vec++ [36]: metapath2vec++ is a heterogeneous information network embedding method based on a meta-path-guided random walk strategy.

For further validation of the impact of target propagation on improving the quality of ADR predictions, we designed a network embedding algorithm without regard for the impact of protein–protein interactions and discarded the PPI dataset; this algorithm was named SDHINE-no-target propagation.

The subsequent ADR prediction methods after the network embedding stage were all based on the same loss function in every prediction task.

Evaluation: We evaluated and compared these algorithms using a 10-fold cross-validation methodology. We randomly selected a fixed percentage (10%) of drugs as the test set and moved all ADRs associated with these drugs from the dataset. The side effects and DDIs of these drugs were all set to 0. The other 90% of the drugs were further divided into the training set and validation set. The training set was formed with 95% of the remaining drugs and was used to train the model. The validation set was formed with the other 5% of the drugs and was used to test the model performance. The independent validation experiments were repeated 30 times with different random divisions of the data for the three sets.

The metrics used to evaluate the model performance were two common ranking metrics: mean average precision at K (MAP@K) and area under the receiver operating characteristic curve (ROC-AUC).

Average precision at K (AP@K) reflects the accuracy of the top-ranked ADRs by a model and can be computed as the mean of Precision@k for each drug or drug pair in the test set. The formula for computing AP@K is given as follows:

(15) $$AP@K=\sum_{k=1}^{K}\mathrm{Precision}(k)/\min\left(L,K\right)$$

where Precision(*k*) is the precision at cut-off *k* in the return list. *L* is the total number of true ADRs for the test drug or drug pairs.

### 3.2. Experimental Results

#### 3.2.1. Visualization Results

First, we compared the performances of all network embedding approaches for the visualization task, which aimed to layout the drug HIN in a 2-dimensional vector space. We mapped the representation vectors of drugs obtained from all comparison approaches to a 2D vector space using the t-SNE [37]. Once a drug is successfully developed, the chemical substructure is fixed. The targets and side effects of a drug are all affected by the chemical substructures of the drug. Therefore, to compare the dimensional reduction performance of different network embedding approaches, the drugs are firstly clustered into different clusters based on their chemical substructures.

**Figure 6:**
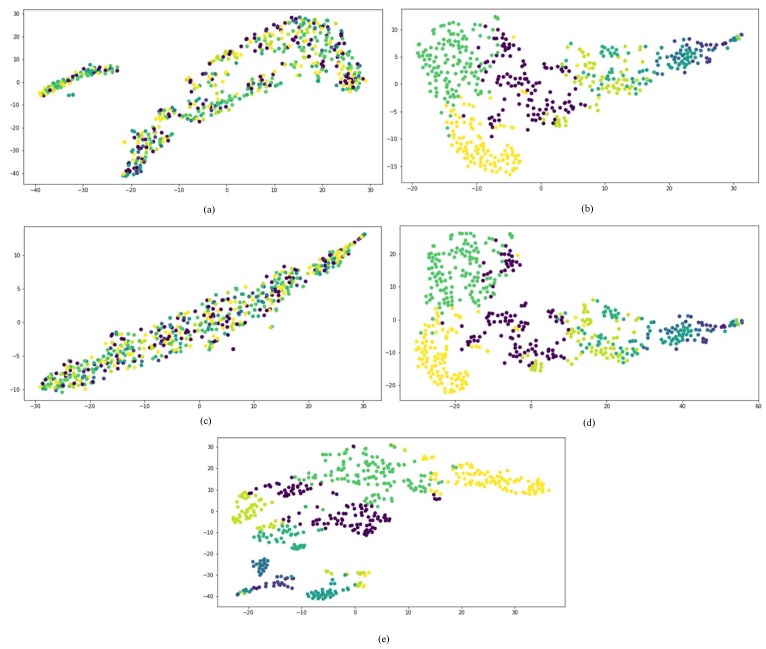
Visualization of the different representations: (**a**) concatenate drug features; (**b**) GraphCNN; (**c**) metapath2vec++; (**d**) SDHINE-no-target propagation; (**e**) SDHINE.

The results are shown in Figure 6, in which drugs belonging to the same cluster are represented by the same color. The concatenated drug features method and metapath2vec++ could not separate drugs from different groups. GraphCNN and SDHINE-no-target propagation basically separated drugs from different groups, but some dark green points were mixed with the other groups. The results obtained with SDHINE were the best among these methods, because it separated most of the drugs from the different groups. This result was consistent with the fact that deep integration of different characteristics can effectively eliminate noise from data and recover the original signal.

#### 3.2.2. Prediction Results

Our experiments further evaluated the drug embeddings obtained through different network embedding methods on different tasks, including side effect predictions for a single drug, binary predictions of the occurrence of DDIs, and multi-label predictions of specific DDI types.

- Task 1: Predicting side effects of a single drug.

To demonstrate the side effect prediction performance based on our network embedding approach, we performed comparison experiments with the aforementioned three baselines and our two proposed models. Predicting the types of side effects caused by one single drug can be formulated into a multi-label classification problem. The output value *y* of the prediction formulation in Section 2.4 is a column vector with 1318 dimensions, and $W^{P}$ is a weighted matrix. Each type of side effect was trained one by one. The negative sampling method was adopted to settle the sample unbalanced problem.

Detailed comparisons of the experimental results are shown in Table 4. Our model based on target propagation clearly performed best compared with other models without a target propagation process in terms of MAP@20 and MAP@100. It was also very close to the best result in terms of MAP@50. Analogously, our approach improved ROC-AUC by 5.87% (84.07% vs. 78.20%) compared to the worst result. From the perspective of the approaches based on deep architecture, GraphCNN and the two SDHINE models performed better than the other models. Meanwhile, the model based on target propagation clearly improved the performance by 3.86% (84.07% vs. 80.21%) compared with the similar model without a target propagation process in terms of ROC-AUC.

- Task 2: Binary prediction of the occurrence of DDIs.

When one drug is administered with another drug, the effect of the drug may be changed, and an unknown side effect may be caused by the DDI. Detecting the occurrence of DDIs is preparation for further research on the ADRs induced by DDIs. When predicting the occurrence of a DDI without regard for the type of DDI, the prediction task can be modeled as a binary classification problem. In this situation, a probability value can be the output layer of the prediction formulation in Section 2.4. $W^{P}$ can be written as a weighted vector. The input layer is formed by the embedding vectors of the two drugs. Table 5 shows a detailed comparison of the experimental results obtained from the binary prediction task of the occurrence of DDIs. The model based on target propagation performed better compared with the similar model without a target propagation process in terms of the mean average precision at *k* and ROC-AUC. The target propagation strategy and deep architecture were still useful for improving the prediction of DDI occurrence.

- Task 3: Multi-label prediction of specific adverse DDI types.

Compared with the prediction of DDI occurrence, most often, we need to address which types of side effects are caused by the DDI. This issue is a multi-label classification problem in which the output layer *y* in the prediction formulation is a column vector with 1318 DDI events. The input layer is concatenated by two drug representation vectors, and $W^{P}$ is a weighted matrix.

Detailed comparison experimental results for specific adverse DDI type identification tasks are shown in Table 6. The model based on target propagation and deep architecture was superior to the models without a target propagation process or deep architecture in terms of not only the mean average precision at *k*, but also the ROC-AUC value.

Based on the results of the three prediction tasks, the network embedding approach with target propagation performance was superior to the approaches without target propagation processing. Moreover, approaches based on deep architecture performed better than the other linear network embedding methods and the combination methods. This result indicates the feasibility of predicting ADRs based on target propagation and proves that the deep learning process is effective at heterogeneous information network embedding.

### 3.3. Performance Comparison of Different Embedding Dimensions

**Figure 7:**
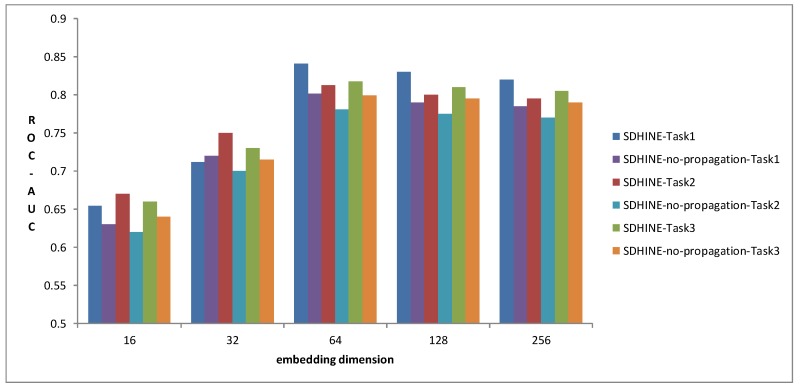
Performance comparison of different embedding dimensions.

To examine the impact of embedding size on prediction performance, we compared SDHINE and SDHINE-no-propagation with different dimensions of drug embeddings for three prediction tasks in terms of ROC-AUC. The results are shown in Figure 7. The prediction performances gradually increased with the increase of embedding dimension and reached the top when embedding dimensions were 64. The prediction performances at 256 dimensions were worse than that at 64 dimensions. This is because the higher dimensional embedding reduced the drug’s differentiability. From the results, we also can find that SDHINE performed better than the same model without the target propagation process at the same embedding dimension on all three prediction tasks. It further verified our assumption that target propagation based on PPI can improve ADRs’ prediction performance.

### 3.4. Case Studies

We examined how the proposed network embedding method predicted potential unknown side effects based on learned drug embeddings. In this article, we only can query the drugs that are in the selected datasets by inputting their id. We took triamcinolone, which is an intermediate-acting synthetic glucocorticoid given orally, by muscular or intra-articular injection, or as a topical ointment or cream and is used to treat various medical conditions (e.g., eczema and ulcerative colitis), as an example. It has been confirmed that it may cause many kinds of side effects, such as cough, headache, influenza, and so on. In the SIDER database, triamcinolone may cause 147 kinds of side effects, and 37 of them have been confirmed in preclinical in vitro safety profiling and clinical drug safety trials.

Table 7 shows the top 10 side effects of triamcinolone based on our model. We found from the results that most of the returned side effects were confirmed and that only two side effects were not confirmed. We analyzed the reasons for this result by taking eye redness as an example. First, we analyzed the target protein of triamcinolone from the DrugBank database and found that triamcinolone activated the target protein NR3C1. Then, we calculated the similarity based on the target propagation meta-path and found that NR3C1 might walk to protein EGFR with a high transition probability in the PPI network. EGFR is the only target protein of the drug gefitinib. We searched the side effects of gefitinib in the SIDER and OFFSIDES databases and found that gefitinib had an eye redness side effect. Thus, we found that eye redness is a potential side effect of triamcinolone based on the above logical inference. The works in [38,39,40] reported that peribulbar injections of triamcinolone may cause intraocular pressure (IOP) elevation, keratitis, and cataract. It is reasonable to believe that eye redness could be one of the ADRs of triamcinolone.

## 4. Conclusions

In this work, we proposed to utilize the impact of protein–protein interactions on drug targets to improve the prediction performance of adverse drug reactions. We designed a meta-path-based heterogeneous information network embedding approach (SDHINE) to integrate multi-source drug information, especially the PPI network. Different meta-path-based proximity calculation methods are designed for different semantic meta-paths. We adopted a semi-supervised stacked denoising auto-encoder to learn drug embeddings in each type of meta-path and integrated them into a second auto-encoder to learn the final drug embeddings. Extensive experiments were performed to compare our algorithm with several state-of-the-art network embedding methods for three ADR prediction tasks, which demonstrated the effectiveness of SDHINE. We also verified the ability of SDHINE to distinguish side effect types and performed a case study by examining the impact of protein-protein interactions on side effects.

In this work, we only considered the meta-paths in which the start and end nodes are all drugs. In future work, we will investigate how to use meta-paths starting from other objects (e.g., side effect nodes) under the guarantee of rationality and interpretability. As a major issue in ADR prediction, we will also consider how to further enhance the interpretability of prediction methods and results based on the semantics of meta-paths.

## Figures and Tables

**Table 1:** Description of the drug data.PPI, protein–protein interaction.

Data Type	Data	Data Source	Dimension
Chemical	Substructures	PubChem	548 × 881
Biological	Target protein	DrugBank	548 × 695
Phenotypic	Treatment disease	DrugBank	548 × 718
Phenotypic	Side effect	SIDER, OFFSIDES	548 × 1318 (1318 ADR events)
Interaction	DDIs	TWOSIDES	548 × 548 × 1318 (1318 ADR events)
Interaction	PPI	HPRD	9519 × 9519 (37,062 interactions)

**Table 2:** Semantics of link types in the drug heterogeneous information network (HIN).

Link Types	Abbreviated Form	Semantics of Link Types
Drug-Drug	*D-D*	Drug-drug interactions
Drug-Chemical	*D-C*	The chemical substructure of a drug
Drug-Protein	*D-P*	The target protein of a drug
Protein-Protein	*P-P*	Protein-protein interactions
Drug-Disease	*D-$D_{I}$*	The therapeutic effect between a drug and a disease
Drug-Side Effect	*D-$S_{E}$*	The side effect between a drug and a disease

**Table 3:** Meta-paths in drug HIN.

Meta-Paths	Abbreviated Form	Semantics of Meta-Paths
Drug-Drug	*DD*	Drug-Drug interactions (at the drug embedding stage, interaction types are not considered).
Drug-Chemical-Drug	*DCD*	Two drugs have a similar chemical substructure.
Drug-Protein-Drug	*DPD*	Two drugs have the same target protein.
Drug-Protein-…-Protein-Drug	$DP^{\left(n\right)}D$ (*n* ≥ 2)	There are protein-protein interactions between the targets of two drugs. For example, the path $D_{1}P_{1}P_{2}D_{2}$ in Figure 4 indicates that the targets of $D_{1}$ and $D_{2}$ are $P_{1}$ and $P_{2}$, respectively. Meanwhile, there is an interaction between $P_{1}$ and $P_{2}$ (in meta-path $DP^{\left(n\right)}D$, there are n−1 protein-protein interactions).
Drug-Disease-Drug	$DD_{I}D$	Two drugs have the same therapeutic effect.
Drug-Side Effect-Drug	$DS_{E}D$	Two drugs have the same side effect.

**Table 4:** Side effect identification performance comparison.

Models	MAP@20	MAP@50	MAP@100	ROC-AUC
Concatenate drug features	0.5590	0.5475	0.5310	0.7820
GraphCNN	0.6510	**0.6493**	0.6321	0.8190
metapath2vec++	0.5835	0.5760	0.5628	0.7845
SDHINE-no-target propagation	0.6508	0.6416	0.6356	0.8021
SDHINE	**0.6653**	0.6479	**0.6361**	**0.8407**

**Table 5:** DDI occurrence identification performance comparison.

Models	MAP@20	MAP@50	MAP@100	ROC-AUC
Concatenate drug features	0.6122	0.5624	0.5432	0.7409
GraphCNN	0.6874	0.6715	0.6219	0.7918
metapath2vec++	0.6542	0.6326	0.5986	0.7332
SDHINE-no-target propagation	0.6813	0.6718	0.6211	0.7814
SDHINE	**0.7015**	**0.6854**	**0.6328**	**0.8124**

**Table 6:** DDI type identification performance comparison.

Models	MAP@20	MAP@50	MAP@100	ROC-AUC
Concatenate drug features	0.6596	0.6144	0.5045	0.74322
GraphCNN	0.6823	0.6681	**0.6137**	0.7851
metapath2vec++	0.6766	0.6567	0.5118	0.7543
SDHINE-no-target propagation	0.6804	0.6622	0.6119	0.7996
SDHINE	**0.6881**	**0.6745**	0.6126	**0.8175**

**Table 7:** Prediction of the top 10 side effects for triamcinolone based on SDHINE.

Top K	Side Effect	Confirmation
K = 1	headache	yes
K = 2	cough	yes
K = 3	fever	yes
K = 4	eye redness	no
K = 5	sneezing	yes
K = 6	nausea	yes
K = 7	rash	yes
K = 8	fatigue	yes
K = 9	dry skin	no
K = 10	conjunctivitis	yes

## References

[1] Giacomini K.M., Krauss R.M., Dan M.R., Eichelbaum M., Hayden M.R., Nakamura Y. (2007). When good drugs go bad. Nature.

[2] Whitebread S., Hamon J., Bojanic D., Urban L. (2005). Keynote review: In vitro safety pharmacology profiling: An essential tool for successful drug development. Drug Discov. Today.

[3] Kanehisa M., Goto S., Furumichi M., Tanabe M., Hirakawa M. (2010). KEGG for representation and analysis of molecular networks involving diseases and drugs. Nucleic Acids Res..

[4] Knox C., Law V., Jewison T., Liu P., Ly S., Frolkis A., Pon A., Banco K., Mak C., Neveu V. (2011). DrugBank 3.0: A comprehensive resource for ’Omics’ research on drugs. Nucleic Acids Res..

[5] Kuhn M., Campillos M., Letunic I., Jensen L.J., Bork P. (2010). A side effect resource to capture phenotypic effects of drugs. Mol. Syst. Biol..

[6] Li Q., Cheng T., Wang Y., Bryant S.H. (2010). PubChem as a public resource for drug discovery. Drug Discovery Today.

[7] Yamanishi Y., Pauwels E., Kotera M. (2012). Drug side effect prediction based on the integration of chemical and biological spaces. J. Chem. Inf. Model..

[8] Li J., Zheng S., Chen B., Butte A.J., Swamidass S.J., Lu Z. (2016). A survey of current trends in computational drug repositioning. Brief. Bioinf..

[9] Xu B., Shi X.F., Zhao Z.H., Zheng W. (2018). Leveraging Biomedical Resources in Bi-LSTM for Drug Drug Interaction Extraction. IEEE Access.

[10] Vilar S., Tatonetti N.P., Hripcsak G. (2015). 3D Pharmacophoric Similarity improves Multi Adverse Drug Event Identification in Pharmacovigilance. Sci. Rep..

[11] Labute M.X., Zhang X., Lenderman J., Bennion B.J., Wong S.E., Lightstone F.C. (2014). Adverse drug reaction prediction using scores produced by large-scale drug-protein target docking on high-performance computing machines. PLoS ONE.

[12] Tatonetti N.P., Ye P.P., Daneshjou R., Altman R.B. (2012). Data-Driven Prediction of Drug Effects and Interactions. Sci. Transl. Med..

[13] Ping Z., Fei W., Hu J. (2014). Towards Drug Repositioning: A Unified Computational Framework for Integrating Multiple Aspects of Drug Similarity and Disease Similarity. AMIA Annu. Symp. Proc..

[14] Zhang W., Chen Y., Tu S., Liu F., Qu Q. Drug side effect prediction through linear neighborhoods and multiple data source integration. Proceedings of the IEEE International Conference on Bioinformatics and Biomedicine.

[15] Segura-Bedmar I. (2011). Using a shallow linguistic kernel for drug–drug interaction extraction. J. Biomed. Inf..

[16] Jin B., Yang H., Xiao C., Zhang P., Wei X., Wang F. Multitask Dyadic Prediction and Its Application in Prediction of Adverse Drug-Drug Interaction. Proceedings of the Thirty-First AAAI Conference on Artificial Intelligence.

[17] Zhang W., Chen Y., Liu F., Luo F., Tian G., Li X. (2017). Predicting potential drug–drug interactions by integrating chemical, biological, phenotypic and network data. BMC Bioinf..

[18] Zitnik M., Agrawal M., Leskovec J. (2018). Modeling polypharmacy side effects with graph convolutional networks. Bioinformatics.

[19] Yan S., Xu D., Zhang B., Zhang H.J., Yang Q., Lin S. (2007). Graph Embedding and Extensions: A General Framework for Dimensionality Reduction. IEEE Trans. Pattern Anal. Mach. Intell..

[20] Cao S., Lu W., Xu Q. Deep neural networks for learning graph representations. Proceedings of the Thirtieth AAAI Conference on Artificial Intelligence.

[21] Huang Z., Mamoulis N. Heterogeneous Information Network Embedding for Meta Path based Proximity. https://arxiv.org/abs/1701.05291.

[22] Li R., Dong Y., Kuang Q., Wu Y., Li Y., Zhu M., Li M. (2015). Inductive matrix completion for predicting adverse drug reactions (ADRs) integrating drug–target interactions. Chemom. Intell. Lab. Syst..

[23] Ma T., Xiao C., Zhou J., Wang F. Drug Similarity Integration Through Attentive Multi-view Graph Auto-Encoders. https://arxiv.org/abs/1804.10850.

[24] Kelley B.P., Sharan R., Karp R.M., Sittler T., Root D.E., Stockwell B.R., Ideker T. (2003). Conserved pathways within bacteria and yeast as revealed by global protein network alignment. Proc. Natl. Acad. Sci. USA.

[25] Yeh C.Y., Yeh H.Y., Arias C.R., Soo V.W. (2012). Pathway Detection from Protein Interaction Networks and Gene Expression Data Using Color-Coding Methods and A^*^ Search Algorithms. Sci. World J..

[26] Codling E.A., Plank M.J., Benhamou S. (2008). Random walk models in biology. J. R. Soc. Interface.

[27] Zou Q., Li J., Song L., Zeng X., Wang G. (2016). Similarity computation strategies in the microRNA-disease network: A Survey. Brief. Funct. Genom..

[28] Shi C., Li Y., Zhang J., Sun Y., Yu P.S. (2016). A Survey of Heterogeneous Information Network Analysis. IEEE Trans. Knowl. Data. Eng..

[29] Shakibian H., Charkari N.M. (2017). Mutual information model for link prediction in heterogeneous complex networks. Sci. Rep..

[30] Chang S., Han W., Tang J., Qi G.J., Aggarwal C.C., Huang T.S. Heterogeneous Network Embedding via Deep Architectures. Proceedings of the 21th ACM SIGKDD International Conference on Knowledge Discovery and Data Mining.

[31] Katz L. (1953). A new status index derived from sociometric analysis. Psychmetrika.

[32] Wang D., Cui P., Zhu W. Structural Deep Network Embedding. Proceedings of the 22nd ACM SIGKDD international conference on Knowledge discovery and data mining.

[33] Kingma D.P., Ba J. Adam: A Method for Stochastic Optimization. https://arxiv.org/abs/1412.6980.

[34] Kipf T.N., Welling M. Semi-Supervised Classification with Graph Convolutional Networks. https://arxiv.org/abs/1609.02907.

[35] Kipf T.N., Welling M. Variational Graph Auto-Encoders. https://arxiv.org/abs/1611.0730821.

[36] Dong Y., Chawla N.V., Swami A. In metapath2vec: Scalable Representation Learning for Heterogeneous Networks. Proceedings of the ACM SIGKDD International Conference on Knowledge Discovery and Data Mining.

[37] Maaten L.V.D., Hinton G. (2008). Viualizing data using t-SNE. J. Mach. Learn. Res..

[38] Hashizume K., Nabeshima T., Fujiwara T., Machida S., Kurosaka D. (2009). A case of herpetic epithelial keratitis after triamcinolone acetonide subtenon injection. Cornea.

[39] Suarez-Figueroa M., Contreras I., Noval S. (2006). Side-effects of triamcinolone in young patients. Arch. Soc. Esp. Oftalmol..

[40] Chew E.Y., Glassman A.R., Beck R.W. (2011). Ocular side effects associated with peribulbar injections of triamcinolone acetonide for diabetic macular edema. Retina.

